# Metformin: Diverse molecular mechanisms, gastrointestinal effects and overcoming intolerance in type 2 Diabetes Mellitus: A review

**DOI:** 10.1097/MD.0000000000040221

**Published:** 2024-10-25

**Authors:** Sami Mohamed

**Affiliations:** a Department of Clinical Sciences, Dubai Medical University, Dubai, United Arab Emirates.

**Keywords:** extended release formulation, glucagon like peptide 1, gut microbiota, Metformin intolerance, type 2 diabetes mellitus

## Abstract

Metformin, the first line treatment for patients with type 2 diabetes mellitus, has alternative novel roles, including cancer and diabetes prevention. This narrative review aims to explore its diverse mechanisms, effects and intolerance, using sources obtained by searching Scopus, PubMed and Web of Science databases, and following Scale for the Assessment of Narrative Review Articles reporting guidelines. Metformin exerts it actions through duration influenced, and organ specific, diverse mechanisms. Its use is associated with inhibition of hepatic gluconeogenesis targeted by mitochondria and lysosomes, reduction of cholesterol levels involving brown adipose tissue, weight reduction influenced by growth differentiation factor 15 and novel commensal bacteria, in addition to counteraction of meta-inflammation alongside immuno-modulation. Interactions with the gastrointestinal tract include alteration of gut microbiota, enhancement of glucose uptake and glucagon like peptide 1 and reduction of bile acid absorption. Though beneficial, they may be linked to intolerance. Metformin related gastrointestinal adverse effects are associated with dose escalation, immediate release formulations, gut microbiota alteration, epigenetic predisposition, inhibition of organic cation transporters in addition to interactions with serotonin, histamine and the enterohepatic circulation. Potentially effective measures to overcome intolerance encompasses carefully objective targeted dose escalation, prescription of fixed dose combination, microbiome modulators and prebiotics, in addition to use of extended release formulations.

**Key points**

• Through AMPK dependent and independent pathways, Metformin inhibits hepatic gluconeogenesis, enhances lipolysis within brown adipose tissue, induces weight loss via GDF15 and counteracts meta-inflammation.
• Within the gut, Metformin beneficially alters microbiota, enhances glucose uptake by enterocytes via GLUT2, increases GLP-1 levels and alters bile acid absorption.
• Metformin intolerance is influenced by dose titration, formulation, gut microbiota, OCT1 inhibition, 5-HT transport, histamine production, bile acid pooling and specific blood-based epigenetic markers.
• Promising interventions to overcome intolerance include tailored dose titration, microbiome modulators, prebiotics and XR formulations.

## 1. Introduction

Metformin, the widely recognized Biguinide, is a Guinide derivative derived from Galegine, which was historically extracted from a plant named Gallega officinalis.^[1–3]^ It is used for treatment of type 2 diabetes mellitus (T2DM), whose incidence is increasing yearly. According to the International Diabetes Federation Diabetes Atlas, nearly half a billion patients are diagnosed with diabetes worldwide, placing a substantial burden on individuals and healthcare systems.^[4]^ Metformin has been, and remains, the first line treatment for T2DM with supportive evidence-based guidelines from relevant authorities, namely the American Diabetes Association and the National Institute for Clinical and Health Excellence.^[5]^

### 1.1. Efficacy in T2DM treatment

Efficacy of Metformin has been proven in treatment of T2DM by enforcing glycaemic control through various mechanisms. Most notably is the glycemic down regulation through inhibition of gluconeogenesis.^[6]^ Additionally, numerous studies examined its impact on the prevention of microvascular and macrovascular complication associated with T2DM.^[7,8]^ Microvascular complications including nephropathy, retinopathy, and neuropathy often occur as result of poorly controlled long-standing disease. However, evidence suggests that Metformin use is linked with substantial risk reduction and decreased incidence.^[9–11]^ Similar association was found for macrovascular complications including cardiovascular, cerebrovascular and peripheral vascular disease.^[12,13]^ Furthermore, recent trials explored its role in diabetes prevention,^[13,14]^ particularly in patients with prediabetes and at-risk populations, though research on this is still developing.

### 1.2. Alternative and novel roles beyond T2DM

Therapeutic uses of Metformin extend beyond T2DM.^[15–18]^ Use in polycystic ovary syndrome, gestational diabetes, nonalcoholic fatty liver disease and nonalcoholic steatohepatitis has been established. Moreover, role of Metformin in cancer prevention and suppression was thoroughly studied.^[19]^ The key aspect by which this is achieved lies in its link to a particular protein kinase named liver kinase B1 (LKB1), which was previously established as a tumor suppressor.^[20]^ LKB1 is required by Metformin to carry its action in the liver by facilitating the activation of the adenosine monophosphate (AMP) activated protein kinase (AMPK),^[21]^ which is responsible for glucose uptake by muscle tissue leading to lowering of blood glucose level.^[22,23]^ This pharmacological pathway that utilizes AMPK was found to affect lipid metabolism as well.^[24]^ Furthermore, Metformin was found to play a role in reduction of tumorogenesis via inhibition of mitochondrial complex I of cancer cells,^[25]^ suggesting a significant role in cancer prevention. Additionally, the use of Metformin in the treatment of inflammatory bowel disease demonstrated positive effects through its action on inflammatory pathways, intestinal barrier integrity and gut microbiota.^[26]^ Metformin also increases the relative abundance of Lactobacillus and Akkermansia species alleviating gut dysbiosis, colonic inflammation and mucus barrier disruption induced by experimental colitis.^[27]^

## 2. Methods

This narrative review was structured and written following the Scale for the Assessment of Narrative Review Articles. Literatures search was conducted through searching Scopus, PubMed and Web of Science databases, including all recently published peer-reviewed articles related to Metformin mechanism of action, gastrointestinal effects and intolerance. As recent observations and studies highlighted new evidence pertaining to diversity of its mechanisms, this article holistically presented the narrative linking Metformin to gastrointestinal effects and intolerance, referencing available evidence. Ethical approval and consent were not required as no experimentation on human subjects or animals were conducted.

## 3. Mechanism of action

### 3.1. Pharmacokinetics

Metformin is absorbed from the gastrointestinal tract, reaching peak plasma concentrations within 2 hours, with a half-life of 2 to 6 hours. It is eliminated by the kidneys and excreted in the urine. Biodistribution of Metformin is dependent on specific cationic compound transporters. These include organic cation transporters and the plasma membrane monoamine transporter.^[28]^ The 3 subtypes of organic cation transporter (OCT 1–3) are expressed in various tissues. Those present in the liver and intestinal epithelial cells facilitate transportation of Metformin into the interstitium.^[29]^ These effects play a role in the resultant plasma concentration of ~4 to 15 μM (~0.5–2.0 μg/mL). However, the optimum concentration required to reach a desired therapeutic effect was previously postulated and yet to be established.^[30]^

### 3.2. Pharmacodynamics

**Figure 1.**
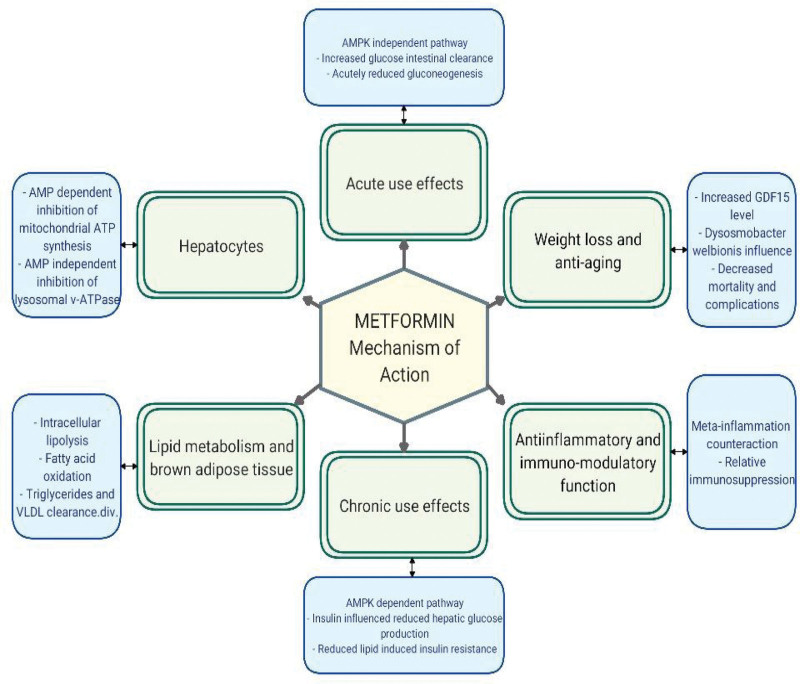
A diagram summarizing diversity of Metformin mechanism of action.

**Figure 2.**
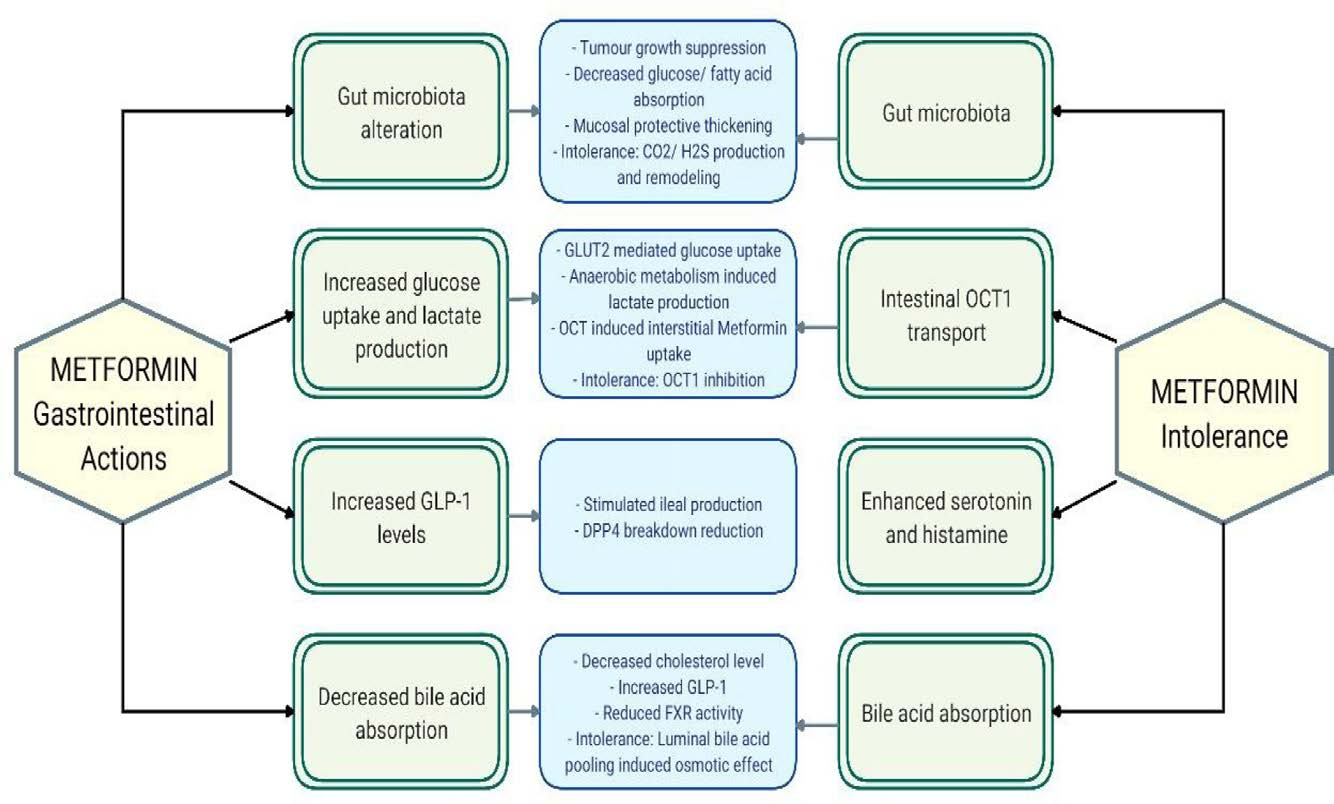
A diagram outlining Metformin related gastrointestinal effects and their possible association with Metformin intolerance.

The primary mechanism of action of Metformin revolves around inhibition of gluconeogenesis by activating AMPK in the liver, eventually lowering blood glucose level.^[31]^ This effect is also achieved through facilitation of glucose uptake by peripheral tissues, especially muscle cells, via increased translocation of glucose transporter proteins 4, which increases insulin sensitivity.^[32]^ Moreover, Metformin exerts its effect either through AMPK dependent or independent mechanisms, and AMPK activation can be achieved even with low concentrations.^[33]^ Furthermore, Metformin reduces intestinal absorption of glucose, adding to its glucose lowering effect. It has been found that Metformin affects the level of glucagon like peptide level 1 (GLP-1), which is responsible for adjusted enhancement of insulin secretion, with involvement of dipeptidyl peptidase-4 released from the gastrointestinal tract.^[34]^ Various mechanisms have been reported through which Metformin exerts its effects (Fig. 1), with special emphasis on the gastrointestinal tract (Fig. 2).^[35,36]^

### 3.3. Effects of acute versus chronic use

Acute use of Metformin significantly affects glycemic control through intestinal clearance. This is exerted regardless of AMPK mediated change in colonic permeability.^[36,37]^ Furthermore, Metformin can acutely decrease hepatic gluconeogenesis.^[38]^ It is relevant to mention that these intestinal, musculoskeletal and hepatic related acute effects were found to take place independently of the LKB1/AMPK pathway.

On the other hand, chronic Metformin use was demonstrated to improve insulin ability to lower hepatic glucose production, positively affecting glycemic control. In addition, lipid lowering effects and reduction of lipid induced insulin resistance also occur. Both effects were shown to be AMPK dependant.^[39,40]^ Moreover, mechanisms through which chronic Metformin use can lower cholesterol where thought to be related to Metformin induced reduction of bile acid absorption.^[41]^

### 3.4. Effects on hepatocytes

As mentioned previously, Metformin effects on the liver can be AMPK dependent or AMPK independent. Moreover, AMPK activation occurs through AMP dependent, or AMP independent pathways factored by Metformin concentration and target organelles within hepatocytes, namely mitochondria and lysosomes.^[42–44]^ Metformin indirectly inhibits mitochondrial adenosine triphosphate (ATP) synthesis through reversible inhibition of respiratory chain complex 1, in addition to subsequent increase in intracellular AMP. Therefore, reduction of gluconeogenesis occurs as it requires ATP abundance.^[45,46]^ Conversely, Metformin affects lysosomes through AMP independent AMPK activation. This effect can be achieved with low Metformin concentrations by inhibiting vacular ATPase (v-ATPase) within lysosomes.^[44,47]^

### 3.5. Effects on lipid metabolism and brown adipose tissue

Studies have shown that brown adipose tissue (BAT) positively contribute to glucose regulation, and thus plays a role in management and prevention of T2DM.^[48]^ Metformin uptake by BAT affects brown adipocyte metabolism, which indirectly contribute to blood lipid profile improvement through enhanced clearance of very low density lipoproteins and triglycerides.^[49,50]^ Moreover, Metformin induced AMPK, and hormone sensitive lipase, activation leads to intracellular lipolysis and mitochondrial fatty acid oxidation within BAT.^[51]^

### 3.6. Effects on weight loss and aging

Metformin use was found to be associated with increased levels of growth differentiation factor 15, which is a stress responsive cytokine of the transforming growth factor-β family, affecting the intestine, skeletal muscles and the liver.^[52]^ Growth differentiation factor 15 is in turn associated with Metformin induced weight loss in patients with T2DM independently of its intended glycemic effects. However, this effect was not confirmed for those with prediabetes and obesity, and warrants further investigation.^[53,54]^ Furthermore, a recently identified butyrate producing bacterium named *Dysosmobacter welbionis*, which is considered a normal commensal, was suggested to be influenced by Metformin treatment, and is associated with reduced body weight, in addition to some beneficial effects on insulin secretion, particularity in obese patient with T2DM.^[55]^

Metformin use has been found to positively affect survival through decreased mortality, particularly of cancer and cardiovascular related adverse events.^[56]^ However, other means of antiaging effects are still unclear, though clinical trials such as Metformin in longevity and targeting aging with Metformin proposed to provide some clarification.^[57]^

### 3.7. Anti-inflammatory and immuno-modulatory effects

Anti-inflammatory properties of Metformin, alongside its immuno-modulatory functions, highlights the possibility of its use in various conditions, despite being not fully understood. Anti-inflammatory properties, through both AMPK dependent and AMPK independent mechanisms helps in counteracting meta-inflammation, which refers to obesity induced inflammatory changes affecting adipose tissue and the liver.^[58]^ These changes may lead to insulin resistance and nonalcoholic steatohepatitis in addition to increased risk of infections such as tuberculosis and severe acute respiratory syndrome corona virus 2. Moreover, use of Metformin was suggested to have protective and therapeutic effects in both tuberculosis and COVID-19, despite previously expected risks of lactic acidosis.^[59,60]^ Furthermore, immuno-modulatory actions of Metformin including immunosuppression, has been suggested to have beneficial effects in inflammatory conditions, such as inflammatory bowel disease.^[26]^

## 4. Metformin and the gastrointestinal system

### 4.1. Interaction with microbiota

Emerging evidence suggests that Metformin causes structural and functional changes in gut microbial constituents, leading to beneficial metabolic changes that affect glucose lowering outcomes in patients with T2DM.^[61]^ These effects vary in different populations depending on inherent factors such as ethnicity.^[62]^ However, abundance of specific bacteria, such as Escherichia spp., and decreased levels others has been found to be associated with Metformin use in both individuals with or without T2DM.^[63]^

Furthermore, Metformin induced alteration of gut microbiota was found to have beneficial effects independent of T2DM. These include antitumor effects through suppression of tumor growth.^[64]^ Moreover, decreased intestinal glucose and fatty acid absorption, related to effects on gene expression, in addition to enhancement of intestinal layer protection through mucosal thickening was also suggested.^[27,65]^

### 4.2. Enhancement of glucose uptake and lactate production

Metformin use enhances glucose uptake and utilization, by enterocytes within the intestine, leading to increased lactate production. This has been previously proven by the use of positron emission tomography–computed tomography (PET–CT), which is an imaging modality often used in malignant conditions. Metformin was shown to increase uptake of 18F-fluorodeoxyglucose, a glucose analogue utilized during PET–CT scan, resulting in interference with imaging outcome in gut tumors, and thus warranting withholding of Metformin use before PET–CT.^[66]^ Moreover, Metformin enhanced glucose uptake occurs through recruitment of GLUT2, located within enterocytes, which in turn enhances its uptake. However, this interaction becomes dis-regulated during fasting, especially in obese individuals.^[67]^ Furthermore, increased uptake and utilization of glucose is followed by subsequent increase in plasma lactate. Both the gut and the liver are implicated in Metformin related lactate production, particularly through anaerobic metabolism of glucose.^[68]^

### 4.3. Increased concentrations of glucagon like peptide 1

Metformin use was found to positively affects GLP-1 levels through both direct and indirect mechanisms. GLP-1 is secreted mainly in the ileum from L cells, then rapidly degraded by dipeptidyl peptidase-4, produced by the liver, within the intestine. Studies suggest that Metformin exerts its effect mainly through stimulation of GLP-1 production and secretion either via increased expression of certain colonic precursor proteins, or via AMPK mediated bile acid pool alteration.^[69,70]^ However, reduction of GLP-1 breakdown by indirect influence on dipeptidyl peptidase-4 was also suggested.^[71]^

### 4.4. Alteration of bile acids absorption

Disruption of the enterohepatic circulation of bile salts was found to be influenced by Metformin use. This leads to increased intestinal bile acid pool secondary to reduced absorption from the ileum. Moreover, Metformin induced alteration in bile acid absorption may potentially lower cholesterol levels, in addition to increased GLP-1 concentration.^[41,69]^ These effects were found to be related to AMPK mediated reduction in Farnesoid X receptor activity, which is a bile acid receptor responsible for its hepatic synthesis and secretion.^[70]^

## 5. Metformin intolerance

Metformin use is often associated with a spectrum of adverse effects, mostly related to the gastrointestinal system, varying in presentation (Fig. 2). These effects are usually transient and tend to resolve spontaneously.^[72,73]^ However, many patients experience more significant effects that affect compliance, and ultimately glycemic control. Diarrhea has been considered one of the most commonly reported symptoms, mostly associated with chronic use.^[74–78]^ However, recent studies, including a systematic review conducted in 2022, found nausea and vomiting to be among the most frequently reported adverse effect, particularity occurring either on treatment initiation or with dose escalation.^[73–75]^ Other adverse effects such as abdominal pain, bloating, constipation, decreased appetite and weight loss were also reported.^[72–81]^ Frequency of common Metformin induced gastrointestinal adverse effects are outlined in Table 1.

**Table 1:** Frequency and details of common Metformin induced gastrointestinal adverse effects.

Gastrointestinal adverse effect	Frequency/details
Diarrhea	• One of the commonest adverse effects. • About 60% of patients taking Metformin reported mild to severe degree, often accompanied by discomfort. • A recent study reported an overall incidence of ~13%.
Nausea and vomiting	• Considered one of the most frequently reported symptoms. • Often occur on treatment initiation, or with dose escalation and tend to diminish over time. • A recent study reported an overall incidence of ~10%.
Abdominal pain	• About 50% of patients taking Metformin experience varying degrees of abdominal pain. • A recent study reported an overall incidence of ~6.5%.
Decreased appetite^[72,73,78,79]^	• Commonly encountered. • May contribute to Metformin induced weight loss.
Bloating^[74,76]^	• Frequently encountered. • A recent study reported an overall incidence of ~9%.
Constipation^[73,74]^	• Less frequently encountered. • A recent study reported an overall incidence of ~2%.

Moreover, studies have shown that approximately 25% of patients with T2DM suffer from Metformin related gastrointestinal adverse effects, with around 5% being unable to tolerate Metformin, potentially resulting in decreased physical and mental health related quality.^[82,83]^ Thus, contributing to either nonadherence or physician reluctance to optimal dose titration prescription.^[76]^

The exact mechanism behind these effects is not fully understood. However, several theories were explored suggesting involvement of Metformin related mechanisms that are potentially accentuated by different factors, which can be modifiable, intrinsic or genetic in nature.

### 5.1. Modifiable factors associated with intolerance

Metformin induced gastrointestinal side effects are typically more common during the initial phase of treatment. This period may last few weeks, then severity of symptoms subsequently tends to decrease. Moreover, dose prescription, in addition to type of formulation, play a significant role in said effects incidence. Higher doses, rapid titration and use of standard immediate release, rather than extended release, formulations were found to be associated with increased incidence.^[74,75]^ Furthermore, concurrent use of certain medications such as Lansoprazole, a proton pump inhibitor that inhibit OCT1, has been implicated in enhancement of Metformin concentrations.^[84]^ Thus, potentially resulting in augmented adverse effects.

### 5.2. Role of gut microbiota in intolerance

It has been suggested that gut microbiota potentially mediate Metformin related gastrointestinal adverse effects,^[72]^ particularly through increased production of CO_2_ and H_2_S as a consequence of Metformin induced metabolic remodeling of *Escherichia* spp.^[63]^ Moreover, a recent study found significant association between Metformin related changes in gut microbiota and its intolerance in patients with T2DM, suggesting its implication.^[85]^

### 5.3. Role of intestinal OCT1

It was found that the role of OCT1, which is responsible for changes in Metformin concentration, has significant association with Metformin intolerance through alteration in its function.^[29,86]^ Moreover, a recent study, among patients with T2DM using Metformin, suggested that changes in certain OCT1 genotype functions significantly increases the likelihood of Metformin induced adverse effects. The odds further increased upon introducing OCT1 inhibiting drugs.^[87]^ Therefore, avoiding use of such drugs may potentially limit incidence of Metformin intolerance.

### 5.4. Effects on serotonin and histamine

When serotonin (5-hydroxytryptamine) is released from the intestine, it potentially causes diarrhea, nausea and vomiting resembling in this manner symptoms of Metformin intolerance. Moreover, Metformin was found to have some structural similarities to certain serotonin receptors and may stimulate its release from the duodenum.^[88]^ On the other hand, some studies suggest its implication in alteration of serotonin transport, and thus, contributing to Metformin related gastrointestinal adverse effect.^[89]^ Furthermore, a recent study suggests that Metformin inhibits diamine oxidase, an enterocyte enzyme involved in histamine metabolism.^[89]^ Given that histamine stimulates gut motility, inhibition of such enzyme, therefore, may contribute to Metformin intolerance.

### 5.5. Bile acid effects

The aforementioned Metformin associated reduction in bile acid absorption has been implicated in Metformin induced gastrointestinal adverse effects. Bile acids are absorbed actively in the ileum and passively in the jejunum. Metformin affects its absorption in both sites, particularly with prolonged use.^[41,90]^ This eventually leads to increased luminal bile salt concentration, resulting in an osmotic effect, which may present diarrhea as an adverse effect.

### 5.6. Genetic factors

It has been recently suggested that epigenetic markers could potentially play a key role in predetermination of Metformin intolerance among patents with T2DM. Studies that performed analysis of DNA methylation found that 4 specific sites differed significantly in patients who are Metformin intolerant, by demonstrating greater methylation, than in those who are not.^[91]^ Moreover, these blood-based epigenetic markers may be measured in patients with T2DM at diagnosis, in order to potential predict intolerance. Furthermore, a recent study found, upon genetic testing, that a specific gene variant on the plasma monoamine transporter, which shares functional similarity to OCT1 and is responsible for serotonin transport, was found to be significantly associated with Metformin induced gastrointestinal adverse effects.^[92,93]^

On the other hand, an attempt to determine characteristic phenotypes of Metformin intolerant patients with T2DM was demonstrated in a previous study and found that they were more likely to be left handed, having blood group A or group A Rh positive and having higher ferritin and low density lipoprotein blood levels, without significant association with obesity nor metabolic syndrome characteristics.^[94]^

## 6. Overcoming Metformin related gastrointestinal adverse effects

### 6.1. Dose titration

Metformin intolerance was found to be dose related, particularly with escalation, and often occurs within few days of starting treatment.^[95]^ Identification of target therapeutic concentrations was therefore thought to provide guidance towards tailored prescription. However, a recent systematic literature search found discrepancies in findings related to the optimum therapeutic concentration of Metformin for treatment of T2DM, and suggested objective outcomes, in terms of glycemic control, to be a preferred guidance.^[96]^ Therefore, careful dose titration with close monitoring could potentially limit incidence of Metformin intolerance.

### 6.2. Prescription methods

Prescription of capsules instead of tablets was suggested to potentially reduce incidence of Metformin related gastrointestinal adverse effects. A previous study found significant reduction upon shifting from Metformin tablets to capsules among patients with T2DM.^[97]^ However, relation of these findings to extended release (XR) formulation warrants further investigation.

Furthermore, use of fixed dose combination regimens in T2DM treatment has been found to improve overall adherence to treatment, in addition to reduction in incidence of Metformin related gastrointestinal adverse effect. A previous systematic review compared its incidence in patients using fixed dose combination containing immediate release, with those containing XR formulation, and suggested improved compliance with lower adverse effects profile with those containing XR formulation.^[98]^ Lower number of pills per day played a significant role in this outcome. However, no direct comparison was analyzed between use of EX formulation alone and its use in fixed dose combination.

### 6.3. Microbiome modulators and prebiotics

Emerging evidence suggests that use of gut microbiome modulators may potentially ameliorate Metformin related gastrointestinal adverse effects, given that some of which are attributable to alteration of gut microbiota. A previous crossover study examined this effect in patients with T2DM and found that those who had microbiome modulators combined with Metformin reported fewer adverse effects, in addition to overall improvement in glycemic control.^[99]^

Furthermore, a recent randomized double blind crossover study explored the possible role of prebiotics in shifting of microbiota composition and activity in patient with T2DM on Metformin. Use of prebiotic fiber supplement was found to be associated with modest microbiota alteration, thus suggesting potential benefits towards Metformin intolerance that require further assessment.^[100]^

### 6.4. Role of extended release formulation

A novel XR formulation of Metformin was developed using GelShield Diffusion System technology in order to overcome the limitations of the standard immediate release formulation which necessitate multiple dosing daily regimen, in addition to the increased need for countering intolerance.^[101]^ The significant role of the XR Metformin maybe attributable to its slower gut absorption coupled, in some instances, with its higher plasma concentrations, while maintaining similar pharmacokinetic parameters.^[102]^ These unique characteristics resulted in significant reduction in Metformin related gastrointestinal adverse effects and a subsequent increase in treatment adherence.^[103]^

Moreover, a previous retrospective cohort study compared Metformin intolerance between patients with T2DM taking immediate release and those taking XR. The frequency of Metformin induced gastrointestinal adverse effects significantly decreased in patients who switched to XR Metformin, reaching less than half the incidence reported when they were taking immediate release formulation.^[104]^ Furthermore, the potential tolerability of XR Metformin was found to be affected by neither obesity nor prescribed dose,^[105]^ while maintaining an equal efficacy when compared to immediate release formulation.^[106]^

## 7. Conclusion

The diversity of mechanisms upon which Metformin carries out its actions, alongside its evident quintessential role in T2DM treatment, sheds lights towards the inevitable need for consistent studying of aspects related to its effects, particularly on interaction with different organic transports, lipid metabolism, aging and anti-inflammatory functions.

Furthermore, the presence of a relatively overlapping link between factors influencing Metformin intolerance and parts of its various effects on the gastrointestinal system, draws attention to the possibility of a better understanding of the process, despite limited exhibition of direct association. However, further evaluation of available ameliorating options, in addition to development of novel approaches, is built upon such understanding.

Metformin prescription and follow-up for patients with T2DM should accompany proper education and counseling on its possible gastrointestinal related adverse effects. Management of which should then follow patient-centered evidence-based approach, utilizing possible options, while attempting to identify inherent predisposing factors, before deciding on discontinuation.

## Acknowledgments

Special appreciations towards Dubai Medical University, and Nile University Sudan for encouraging research and publication.

## Author contributions

**Conceptualization:** Sami Mohamed.

**Investigation:** Sami Mohamed.

**Methodology:** Sami Mohamed.

**Project administration:** Sami Mohamed.

**Supervision:** Sami Mohamed.

**Validation:** Sami Mohamed.

**Visualization:** Sami Mohamed.

**Writing – original draft:** Sami Mohamed.

**Writing – review & editing:** Sami Mohamed.
